# Supplementary material for: COZOID: contact zone identifier for visual analysis of protein-protein interactions
Source: BMC Bioinformatics. 2018 Apr 6;19:125. doi: 10.1186/s12859-018-2113-6 (PMC5889581; doi:10.1186/s12859-018-2113-6)
Supplement: Supplementary file 2 — Software build. Executable binary file of the software tool. (ZIP 118,784 kb) [file 12859_2018_2113_MOESM2_ESM.zip › cozoid/java/docs/beansbinding-1.2.1-doc.zip!/org/jdesktop/observablecollections/package-use.html]

Uses of Package org.jdesktop.observablecollections (Beans Binding)


---


|  |  |  |
| --- | --- | --- |
| **Overview** \| **Package** \| Class \| **Use** \| **Tree** \| **Deprecated** \| **Index** \| **Help** | |  |
| PREV   NEXT | **FRAMES**    **NO FRAMES**     **All Classes** |  |


---


## **Uses of Package org.jdesktop.observablecollections**

| Packages that use org.jdesktop.observablecollections | |
| --- | --- |
| **org.jdesktop.observablecollections** | Provides support for observing changes to collection classes. |
| **org.jdesktop.swingbinding.impl** | Private implementation details for Swing binding. |

Classes in org.jdesktop.observablecollections used by org.jdesktop.observablecollections

****ObservableCollections.ObservableListHelper****
  
          `ObservableListHelper` is created by `observableListHelper`,
and useful when changes to individual elements of the list can be
tracked.

****ObservableList****
  
          A `List` that notifies listeners of changes.

****ObservableListListener****
  
          Notification types from an `ObservableList`.

****ObservableMap****
  
          A `Map` that notifies listeners of changes to the `Map`.

****ObservableMapListener****
  
          Notification types from an `ObservableMap`.

Classes in org.jdesktop.observablecollections used by org.jdesktop.swingbinding.impl

****ObservableList****
  
          A `List` that notifies listeners of changes.

****ObservableListListener****
  
          Notification types from an `ObservableList`.

---


|  |  |  |
| --- | --- | --- |
| **Overview** \| **Package** \| Class \| **Use** \| **Tree** \| **Deprecated** \| **Index** \| **Help** | |  |
| PREV   NEXT | **FRAMES**    **NO FRAMES**     **All Classes** |  |


---
